# Withdrawal from Cocaine Self-Administration Alters NMDA Receptor-Mediated Ca^2+^ Entry in Nucleus Accumbens Dendritic Spines

**DOI:** 10.1371/journal.pone.0040898

**Published:** 2012-08-03

**Authors:** Carrie R. Ferrario, Ivan Goussakov, Grace E. Stutzmann, Marina E. Wolf

**Affiliations:** 1 Department of Pharmacology, University of Michigan, Ann Arbor, Michigan, United States of America; 2 Section of Neurology, Department of Pediatrics, The University of Chicago, Chicago, Illinois, United States of America; 3 Department of Neuroscience, Rosalind Franklin University of Medicine and Science, North Chicago, Illinois, United States of America; Neuroscience Campus Amsterdam, VU University, The Netherlands

## Abstract

We previously showed that the time-dependent intensification (“incubation”) of cue-induced cocaine seeking after withdrawal from extended-access cocaine self-administration is accompanied by accumulation of Ca^2+^-permeable AMPA receptors (CP-AMPARs) in the rat nucleus accumbens (NAc). These results suggest an enduring change in Ca^2+^ signaling in NAc dendritic spines. The purpose of the present study was to determine if Ca^2+^ signaling via NMDA receptors (NMDARs) is also altered after incubation. Rats self-administered cocaine or saline for 10 days (6 h/day). After 45–47 days of withdrawal, NMDAR-mediated Ca^2+^ entry elicited by glutamate uncaging was monitored in individual NAc dendritic spines. NMDAR currents were simultaneously recorded using whole cell patch clamp recordings. We also measured NMDAR subunit levels in a postsynaptic density (PSD) fraction prepared from the NAc of identically treated rats. NMDAR currents did not differ between groups, but a smaller percentage of spines in the cocaine group responded to glutamate uncaging with NMDAR-mediated Ca^2+^ entry. No significant group differences in NMDAR subunit protein levels were found. The decrease in the proportion of spines showing NMDAR-mediated Ca^2+^ entry suggests that NAc neurons in the cocaine group contain more spines which lack NMDARs (non-responding spines). The fact that cocaine and saline groups did not differ in NMDAR currents or NMDAR subunit levels suggests that the number of NMDARs on responding spines is not significantly altered by cocaine exposure. These findings are discussed in light of increases in dendritic spine density in the NAc observed after withdrawal from repeated cocaine exposure.

## Introduction

The idea that experience can shape or reorganize synaptic connections is a fundamental tenet of neuroscience [1]. During the last decade, a number of studies have found that exposure to drugs of abuse (such as cocaine, amphetamine, methamphetamine and nicotine) can alter dendritic morphology in brain regions implicated in addiction, although the relationship of this structural plasticity to behavioral changes elicited by repeated drug exposure remains unclear [2], [3]. In the nucleus accumbens (NAc), it is well established that the density of dendritic spines on medium spiny neurons (MSN) is increased after withdrawal from either non-contingent or self-administered cocaine e.g., [4]–[8]. Cocaine-induced increases in dendritic branching and changes in spine head morphology have also been observed [4], [5], [9], [10]. Dendritic spine heads are the primary site of glutamatergic synaptic contacts in the NAc [11]. Several studies have provided clues to understanding the mechanisms involved in the development of increased spine density [6], [12]–[16]. However, little is known about functional aspects of the additional spines observed after cocaine exposure, including their content of glutamate receptors, although recent reports suggest that at least some of these spines contribute to synaptic transmission [9], [17].

Excitatory transmission onto MSN of the NAc is critical for cocaine seeking behavior in several rodent models of cocaine addiction [18], [19]. We have focused recently on the “incubation” model. Incubation refers to the progressive intensification of cue-induced cocaine-seeking that occurs during the first month or two of withdrawal from extended access cocaine self-administration [20]. This provides an animal model for increased vulnerability to cue-induced relapse in human addicts following a period of forced abstinence due to incarceration or hospitalization [21]. We previously showed that the expression of incubated cue-induced cocaine seeking on withdrawal day (WD) 45 is mediated by activation of Ca^2+^-permeable AMPA receptors (CP-AMPARs) in the NAc [22]. CP-AMPARs contribute very little to excitatory transmission in the NAc of drug-naïve rodents or rodents given repeated non-contingent cocaine injections, but accumulate in NAc synapses during withdrawal from extended access cocaine self-administration [22]–[27]. The synaptic incorporation of CP-AMPARs is very persistent, as it can be detected as early as WD30 [26] and continues at least through WD70 [28].

These results suggest an enduring change in Ca^2+^ signaling in MSN in association with the incubation of cocaine craving due to synaptic incorporation of CP-AMPARs. The purpose of the present study was to determine if Ca^2+^ signaling via NMDA receptors (NMDAR) is also altered after incubation. We tested this by examining two aspects of NMDAR transmission in the NAc after 45 days of withdrawal from either saline or cocaine self-administration: 1) NMDAR-mediated Ca^2+^ entry in individual MSN spine heads and corresponding NMDAR-mediated somatic currents elicited by glutamate uncaging, and 2) NMDAR subunit levels in a postsynaptic density (PSD) fraction.

## Materials and Methods

### Ethics Statement

All procedures were approved by Rosalind Franklin University of Medicine and Science Institutional Animal Care and Use Committee, in accordance with AAALAC and NIH guidelines, under protocol numbers 09–14 and 11–14.

### Self-administration

Procedures were conducted as previously described [22]. Briefly, male Sprague Dawley rats (250–275 g on arrival) were implanted with a jugular catheter and allowed to self-administer cocaine (0.5 mg/ kg/32 μl) or saline (32 μl/infusion) during 10 daily sessions, each lasting 6 h (Coc-SA and Sal-SA groups, respectively). After 45 days of withdrawal in their home cages, rats were decapitated and tissue was obtained for either Ca^2+^ imaging studies (Sal-SA N = 5 rats, Coc-SA N = 3 rats) or preparation of a PSD fraction (Coc-SA N = 7 rats, Sal-SA N = 9 rats).

### Slice preparation and electrophysiology

Coronal slices (300 µm) containing the NAc were prepared as previously described [22]. Slices were transferred to a recording chamber and superfused at 2 ml/min with standard aCSF solution containing (in mM): 125 NaCl, 2.5 KCl, 2 CaCl2, 1.2 MgSO4, 1.25 NaH2PO4, 25.0 NaHCO3, 10 D-dextrose and 0.05 picrotoxin (pH 7.3–7.4; equilibrated with 95% O2 and 5% CO2 at room temperature). Osmolarity was maintained at 300 mOsm. Patch pipettes (4–5 MΩ) were filled with intracellular solution containing (inmM): 135 Cs-methanesulfonate, 10 HEPES, 10 Na-phosphocreatine, 2 MgCl2, 4 NaATP, 0.4 NaGTP, 0.05 bis fura-2 (Invitrogen, Carlsbad, CA), 0.03 ryanodine and 10 mg/ml heparin (pH 7.3). Ryanodine and heparin were added to block Ca^2+^ release from intracellular ryanodine- and IP3 receptor-sensitive stores, respectively. Blockers of voltage-gated Ca^2+^ channels (VGCC) were not included because these channels are predominantly located in somatodendritic regions [29] and activated at more positive membrane potentials than used here (−20 to +20 mV compared to −80 mV used here). In addition, their low channel conductance and rapid inactivation kinetics make it unlikely they would contribute to Ca^2+^ transients under our recording conditions [30]. MSN in the NAc core were identified visually via IR/DIC optics. Whole-cell patch clamp configuration was achieved in voltage clamp mode using a Digidata 1322 AnalogDigital converter and Multiclamp 700 B amplifier. Data were recorded and analyzed using pClamp 10.2 software (Molecular Devices Corp., Union City, CA). Membrane potentials were held at −80 mV in voltage-clamp mode and access resistance was monitored throughout the experiment and maintained below 10 MΩ. The experimental sequence was as follows: After acquiring a whole cell configuration, the slices were perfused with a [0] Mg^2+^ aCSF solution containing CNQX (20 µM) and TTX (1 µM) for 10 min. Several control UV flashes (20 ms) were administered in the absence of caged compounds. Then, the aCSF solution was switched to one including 0.5 mM MNI-caged-L-glutamate (Tocris Biosciences, Ellisville, MO) and 10 μM D-serine, and slices were perfused for 1 min before photo-uncaging using a 20 ms UV flash. The evoked whole cell NMDAR currents and Ca^2**+**^ responses in dendritic spines were captured simultaneously. At the conclusion of each experiment, the specificity of NMDAR responses was confirmed by repeating the photolysis protocol in the presence of APV (50 µM). Unless otherwise specified, reagents were obtained from Sigma-Aldrich (St. Louis, MO).

### 2-photon Ca^2+^ imaging and UV photolysis

We analyzed 60 spines from the Coc-SA group (6 slices prepared from 3 different rats) and 133 spines from the Sal-SA group (9 slices prepared from 5 different rats). Ca^2+^ imaging within individual dendritic spine heads was performed using a video-rate multiphoton imaging system based on an upright Olympus BX51 microscope frame [31]. Laser excitation was provided by trains (80 MHz) of ∼100 fs pulses at 780 nm from a Ti sapphire laser (Mai Tai Broadband, Spectra-Physics, Mountain View, CA). The laser beam was scanned by a resonant galvanometer (General Scanning Lumonics, Waterton, MA) allowing rapid (7.9 kHz) bidirectional scanning in the *x*-axis and by a conventional linear galvanometer in the *y*-axis, to provide a full frame-scan rate of 30 fps. The laser beam was focused onto the tissue through a 40X water-immersion objective (NA = 0.8). Emitted fluorescence light was detected by a wide-field photomultiplier (Electron Tubes Inc, England) to derive a video signal that was captured and analyzed by Video Savant 5.0 software (IO Industries, Ontario, Canada). This procedure was then repeated in another slice (to avoid effects produced by prior glutamate uncaging). Further analysis of background corrected images was performed using Metamorph software. For clarity, pseudocolored images of fura-2 fluorescence are expressed as inverse pseudo-ratios so that increases in [Ca^2+^] correspond to increasing ratios of F0/ΔF (F0 is the average resting fluorescence at baseline, and ΔF is the decrease of fluorescence upon Ca^2+^ release). Data indicating relative percentage changes in fluorescent intensity were calculated as percent over baseline: (F0/ΔF−1) ×100. UV flash photolysis of MNI-caged-L-glutamate was accomplished using an X-Cite 120 Fluorescence Illumination system (Photonic Solution Inc., Canada) and narrow UV filter cube (360–380 nm) in a light path separate from the IR laser input, with exposure time determined by electronic shutters (Uniblitz) operated and synchronized through digital outputs (Digidata 1322 A-D board) controlled by pClamp 10.2 software. Two-dimensional X–Y video-rate data acquisition was used to capture rapid NMDA-evoked Ca^2+^ transients at high spatial and temporal resolution, see [32], [33]. The percentage of spine heads generating an NMDA-mediated Ca^2+^ response within a full image frame was determined by counting the total number of visible spines and determining the fraction of ‘responding’ spines, expressed as ‘% responding’. The threshold for Ca^2+^ responding was determined by a signal temporally synchronized with the UV flash at least 10% over baseline level with onset and decay kinetics distinguishable from background. Groups were compared with a two-tailed t-test (significance set at p<0.05).

### PSD preparation and analysis

A PSD fraction was prepared from bilateral NAc tissue of each rat using a previously described method [26], [34]. NAc tissue was homogenized (Wheaton Potter-Elvehjem Tissue Grinders, Fisher Scientific, Pittsburgh, PA) in HEPES-buffered sucrose (0.32 M sucrose, 4 mM HEPES, pH 7.4) containing 2 mM EGTA, 50 mM NaF, 10 mM PPi, 1 mM NaOV, 1 uM okadaic acid, 1 mM phenylmethyl sulfonyl fluoride (PMSF), 1 uM microcystin-LF and 1X protease inhibitor cocktail set 1 (Calbiochem, Darmstadt, Germany). The homogenate was centrifuged (800× g, 10 min, 4°C) to remove the pelleted nuclear fraction (P1). The resulting supernatant (S1) was centrifuged (10,000× g, 15 min, 4°C) to yield a crude membrane fraction (P2) which was washed and then lysed hypo-osmotically with cold 4 mM HEPES (pH 7.4) with inhibitors and centrifuged (25,000× g, 20 min, 4°C) to yield the LP1 fraction. The LP1 was re-suspended in HEPES-buffered sucrose with inhibitors and run on a discontinuous sucrose gradient (1.2 M, 1.0 M, and 0.8 M sucrose with inhibitors) and centrifuged (125,000×g, 2 h, 4°C). The synaptic plasma membrane (SPM) was collected between 1.0 M and 1.2 M sucrose and diluted 2.5 times in 4 mM HEPES with inhibitors and pelleted by centrifugation (150,000×g, 30 min, 4°C). The SPM pellet was re-suspended in a 0.5% Triton X-100, HEPES-EDTA solution (50 mM HEPES, 2 mM EDTA, pH 7.4 with inhibitors), incubated with rotation (15 min, 4°C) and centrifuged (32, 000×g, 20 min, 4°C) to pellet the insoluble postsynaptic density fraction (PSD). The PSD fraction from each rat was then resuspended in 200 µl of Laemmli sample treatment buffer containing 100 mM dithiothreitol (DTT). Samples were stored at −80°C. SDS-PAGE and immunoblotting were performed as described previously [26]. Equal volumes of each sample were loaded onto gels, with samples from each experimental group evenly distributed on each gel. The following primary antibodies were utilized: NR1 (1∶1000; AB05-432; Millipore, Billerica, MA), NR2B (1∶1000; 454582; Calbiochem-EMD Chemicals, Gibbstown, NJ), and NR2A/B (1∶2500; AB1548W; Millipore). Groups were compared with a two-tailed t-test (significance set at p<0.05).

## Results

**Figure 1:**
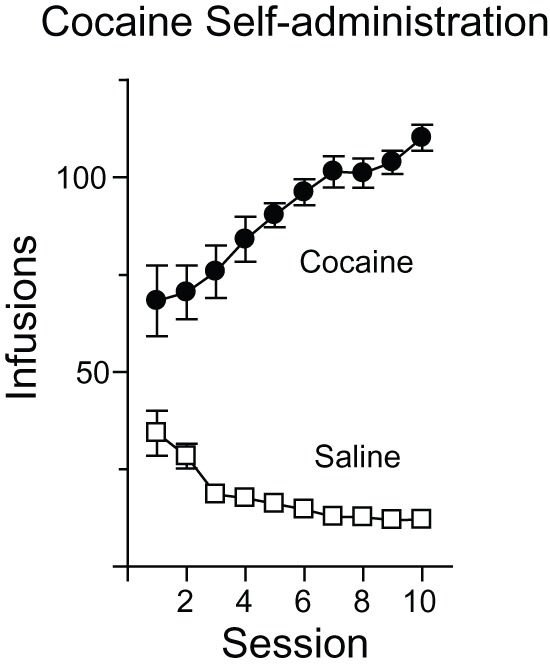
Self-administration behavior. Mean number of infusions (± SEM) taken during each 6 hour self-administration session for saline (open squares) or cocaine (closed circles) groups.

Rats self-administered cocaine for 6 hours per day for 10 days (Coc-SA group), as in our previous studies [22], [26], [27], [35]. All rats readily acquired cocaine self-administration. Control rats self-administered saline (Sal-SA group). Fig 1A shows the average number of infusions of cocaine (closed circles) or saline (open squares) taken per session.

NAc slices were prepared from Coc-SA and Sal-SA rats on WD45–47, and NMDAR function in MSN spines was probed using 2-photon Ca^2+^ imaging combined with whole cell patch clamp recording and UV flash photolysis of caged glutamate. In these experiments, MSN in the NAc core were filled with a Ca^2+^ indicator (bis fura-2) via the patch pipette, caged glutamate was washed into the bath solution and UV flash photolysis was used to release glutamate into the extracellular space. To achieve selective NMDAR activation upon glutamate uncaging, compounds were included in the bath solution and patch pipette to eliminate other sources of Ca^2+^ entry or intracellular Ca^2+^ release (see Methods and Materials). Whole cell NMDAR currents were monitored using patch clamp recordings from the soma, and Ca^2+^ entry through the NMDAR channel was monitored with 2-photon imaging within a dendritic branch containing a number of individual spine heads.

**Figure 2:**
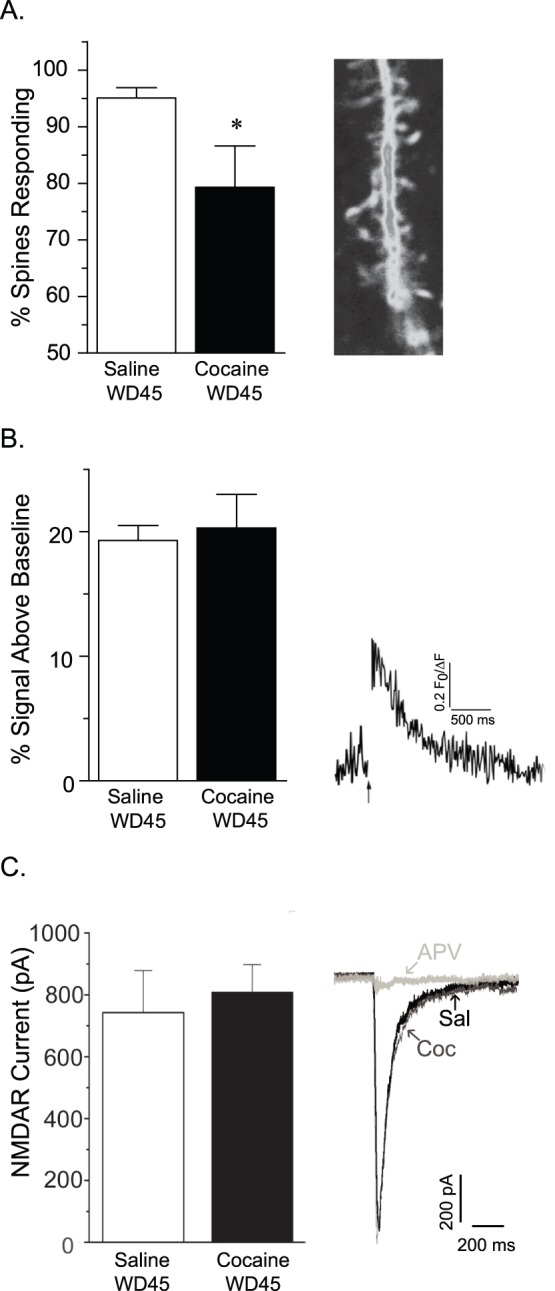
The percentage of spines exhibiting NMDAR-mediated Ca^2+^ entry is decreased after 45 days of withdrawal from cocaine self-administration. A) Left: The percent of dendritic spines showing a NMDAR-mediated Ca^2+^ response upon photolysis of caged glutamate is significantly reduced in the cocaine group compared to the saline group. Right: Representative 2–photon image of a fura–2 filled dendrite from a MSN in the NAc core. B) Left: In spines that did exhibit an NMDAR-mediated Ca^2+^ response, the relative magnitude of the Ca^2+^ response did not differ between saline and cocaine groups. Right: Representative NMDAR-evoked Ca^2+^ transient from a MSN spine from the saline group. C) Left: The peak amplitude of the NMDAR-evoked whole cell currents did not differ between saline and cocaine groups. Right: Representative traces of NMDAR currents in MSN from saline (black) and cocaine (dark gray) groups. NMDAR currents in both groups were completely blocked by addition of APV (light gray trace shows APV blockade in a saline-treated animal). A 20 ms UV flash in the absence of caged MNI-glutamate did not generate a current response (data not shown). Data are presented as mean (± SEM). *p<0.05.

For each brain slice examined in our experiments, we first selected a region of interest containing dendritic spines located on third-order (or greater) processes of MSN, because this is where increased spine density has been detected on WD30 from a nearly identical cocaine self-administration regimen [8] as well as in experiments using non-contingent cocaine at a similar withdrawal time ([4]; WD25). The percent of spine heads generating an NMDAR-mediated Ca^2+^ response within the dendritic branch image frame was determined by counting total spines (based on resting fura-2 fluorescence) and determining the fraction of responding spines (identified by a>10% change in bis fura-2 fluorescence upon MNI-caged-L-glutamate photolysis). We found a significant decrease in the percent of spines responding to NMDAR activation in the Coc-SA group compared to the Sal-SA group (Fig 2A). In those spines that did respond, the magnitude of the Ca^2+^ signal did not differ between cocaine and saline groups (Fig 2B). NMDAR currents were simultaneously recorded throughout glutamate uncaging using whole cell patch clamp recordings. We found that the NMDAR whole cell currents did not differ between groups [Fig 2C; t_(1,14)_ = 0.41; p>0.05, n = 8 in each group]. NMDAR-specificity of the response to glutamate uncaging was confirmed by repeating the photolysis protocol in the presence of the NMDAR antagonist APV (50 µM); as expected, this abolished the evoked whole cell current (Fig 2C) as well as the Ca^2+^ responses (not shown). While we cannot rule out a potential contribution of voltage sensitive Ca^2+^ channels (VSCC) to our signal [36] the most abundant potential contributor in this class of VSSC is the low-threshold, high conductance L-type channel, which is strongly expressed in the somatodendritic region of the neuron [37].

**Figure 3:**
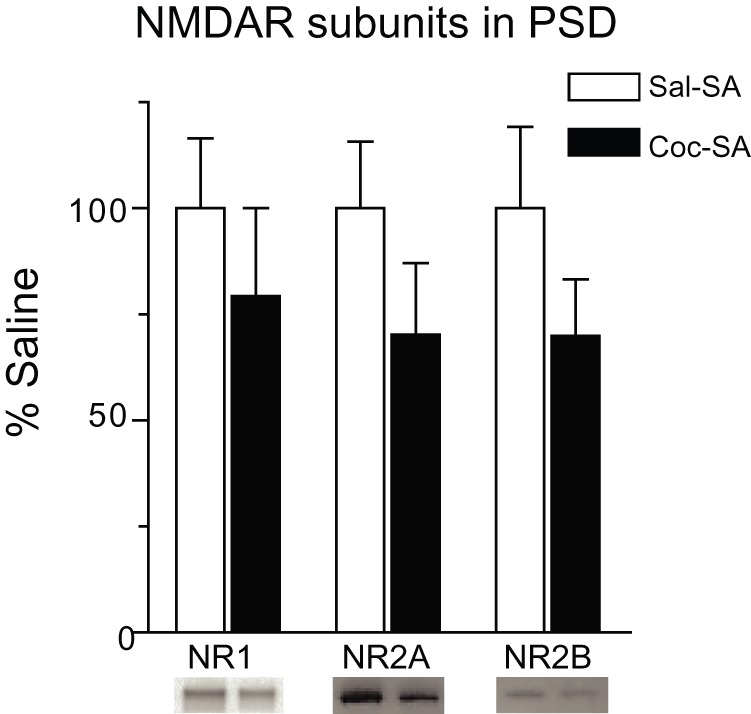
Expression of NMDAR subunits in the postsynaptic density (PSD) fraction after 45 days of withdrawal from cocaine or saline self-administration. Data are presented as mean (± SEM) expressed as percent of saline controls. NR1, NR2A and NR2B protein levels in the cocaine group were slightly decreased on WD45 compared to the saline group, though this did not reach statistical significance. Representative blots are shown below each bar.

The results of our electrophysiological recordings suggest that the overall ability of MSN to respond to NMDAR activation was unchanged in the Coc-SA group. This can be reconciled with the decrease in the proportion of spines showing NMDAR-mediated Ca^2+^ entry by proposing that MSN in the Coc-SA group contain more spines which lack NMDARs (non-responding spines) but that the number of NMDARs on responding spines is not significantly altered by cocaine exposure. If this were the case, we would expect synaptic NMDAR subunit expression to be similar between cocaine and saline self-administering groups. In a prior study, we used a protein crosslinking assay to measure NMDAR surface expression in the NAc of Sal-SA and Coc-SA groups on WD45 and found no significant differences [22]. However, this assay measures both synaptic and extrasynaptic surface NMDAR pools and therefore might have failed to detect a change confined to the synaptic pool. To overcome this problem and more selectively assess synaptic NMDAR levels, we prepared PSD fractions from the NAc of Sal-SA and Coc-SA rats on WD45. Immunoblotting results in the PSD fractions are shown in Fig. 3. We observed no significant group difference in NMDAR subunit levels, although there were trends towards decreased levels of all subunits (NR1, NR2A and NR2B; p = 0.10–0.22) in the Coc-SA group compared to the Sal-SA group.

## Discussion

Increased spine density in the NAc after withdrawal from repeated cocaine exposure has been reported by multiple groups (see Introduction). Relatively little is known about functional aspects of the additional spines, although morphological and electrophysiological results obtained after non-contingent cocaine exposure suggest that they may form functional synapses [9], [17]. In the present study, we photo-released glutamate near MSN spines and monitored both NMDAR-mediated Ca^2+^ entry and electrical responses in the soma. Based on our prior report of increased spine density after a nearly identical cocaine self-administration regimen and withdrawal time [8], we assume that spine density was increased in our Coc-SA group. If there was a corresponding increase in synapses in the Coc-SA group, and the new synapses contained NMDARs, then we should have observed larger NMDAR-evoked currents in the Coc-SA group compared to the Sal-SA group. In fact, whole cell currents did not differ between groups, suggesting that increased spine density in the Coc-SA group is not accompanied by an increase in synaptic NMDARs on processes. More surprisingly, whereas nearly all spines in the Sal-SA group responded to glutamate uncaging with NMDAR-mediated Ca^2+^ entry, we found a significantly smaller percentage of responding spines in the Coc-SA group. We interpret these results to indicate that a greater proportion of spines in the Coc-SA group lack functional NMDARs. A potential alternative explanation is suggested by a prior study showing decreased NMDAR-mediated Ca^2+^ transients in spines with stronger diffusional coupling to the dendrite [38]. However, our results revealed non-responsive spines, rather than less responsive spines, in the cocaine group, and are therefore more consistent with a lack of NMDARs than with an alteration in Ca^2+^ handling. While it would be of interest to determine the morphological features of the spines exhibiting different Ca^2+^ responses, the two dimensional images obtained with Ca^2+^ imaging do not accurately reflect the morphology of the entire spine in three dimensions. Thus, determining the morphological features of the spines exhibiting different Ca^2+^ responses requires other approaches and will be addressed in future studies.

It should be noted that we are not specifically activating NMDARs located within spine heads, but rather photoreleasing glutamate over a region of neuropil. Therefore, the possibility exists that dendritic NMDARs are generating a Ca^2+^ signal that could diffuse into spines, contributing to our results. However, while it is certainly the case that Ca^2+^ from dendritic compartments can invade spine heads through passive diffusion, this diffusion is typically modeled from spine to dendrite, with initial faster decay kinetics in the spine than in the neighboring dendrite [39]. In addition, the spine neck provides a significant barrier to diffusion in either direction, although the extent of the barrier depends upon the spine classification [40], [41].

Biochemical studies of Coc-SA and Sal-SA groups (prepared identically to those used for Ca^2+^ imaging and electrophysiology) found no significant differences in NMDAR subunit protein levels in NAc PSD fractions, although the Coc-SA group showed trends towards decreased NMDAR subunit levels. These results are in general agreement with our electrophysiological results showing no significant changes in NMDAR-mediated currents. Both findings are consistent with the idea that an increase in dendritic spine number (and perhaps synapses; see [9], [17]) is not necessarily accompanied by an increase in NMDAR expression. A caveat regarding our biochemical results is that cocaine increases spine density in specific portions of the dendritic arbor [2], [8], whereas our PSD fraction is prepared from the whole NAc and may therefore be inadequate for detecting spatially restricted changes related to increased spine density. Another consideration is that electrophysiological studies sampled NAc neurons in the core subregion, whereas our PSD fraction was prepared from the whole NAc in order to obtain an adequate yield of protein from each rat.

We have shown that CP-AMPARs, which normally account for ∼5% of the evoked ESPC in the adult rat NAc, accumulate at excitatory synapses in the NAc of Coc-SA rats (using a regimen identical to the present one) and account for ∼30% of the evoked EPSC on WD42–47 [22]. This is a functionally significant neuroadaptation because CP-AMPARs have different properties than Ca^2+^-impermeable AMPARs, including a larger single channel conductance [42], and of course contribute to Ca^2+^ signaling, e.g.,[43]. If CP-AMPARs are added to spines lacking NMDARs, then CP-AMPARs rather than NMDARs would serve as the source of glutamate-induced Ca^2+^ entry. This predicts an inversion of the induction requirements for LTP, since NMDA receptors are activated at depolarized potentials whereas CP-AMPARs are blocked at depolarized potentials by intracellular polyamines, see [44], [45]. Unfortunately, it will be difficult to determine if CP-AMPARs are present in the spines that lack NMDARs as concurrent imaging of NMDAR and AMPAR currents is not reliably feasible within individual spine heads. Moreover, significant challenges exist for detecting and imaging within individual spines the small channel conductance (10–20 pS) of the CP-AMPARs [46], not all of which is attributable to Ca^2+^. A much larger Ca^2+^ conductance is carried through NMDAR channels,>60pS; [47]. Nevertheless, it is intriguing to note that synapses containing CP-AMPARs tend to exhibit low NMDAR currents [44], [45], [48].

The NAc receives glutamate afferents from several regions, including the prefrontal cortex, basolateral amygdala, ventral subiculum, and thalamus, and inputs from these regions may converge on a single MSN, e.g., [11], [49]–[52]. If new cocaine-induced spines with an abnormal complement of ionotropic glutamate receptors are preferentially innervated by a particular set of afferent fibers, this could alter the balance of information flow into the NAc in a manner that promotes cue-induced cocaine seeking.

An alternative hypothesis is that the non-responding spines in the Coc-SA group not only lack NMDARs, but also lack AMPARs and thus do not participate in functional excitatory transmission. According to this hypothesis, CP-AMPARs would be added to existing spines, either at synapses that already possess Ca^2+^-impermeable AMPARs or perhaps at silent synapses (which contain only NMDARs). Interestingly, the number of silent synapses in the NAc increases with non-contingent cocaine exposure but then normalizes during withdrawal, perhaps due to AMPAR addition [53]. It is possible that a similar sequence occurs during withdrawal from extended access cocaine self-administration. Supporting the idea that CP-AMPARs are added to synapses that already contain NMDARs, there is evidence that NMDARs are present at synapses prior to the addition of AMPARs during normal development and during the unsilencing of silent synapses [54], [55]. On the other hand, there is no reason to assume that cocaine-induced spine plasticity recapitulates normal development or normal plasticity. Furthermore, there is precedent for “AMPAR-only” synapses on neurons that express NMDARs [56].

Non-contingent cocaine exposure can produce different plasticity in NAc MSN expressing D1 versus D2 dopamine receptors [9], [12], [57], although this is not always the case, e.g., [58], [59]. In the incubation model, no evidence for heterogeneous responding exists – in fact, all of the adaptations in excitatory transmission that we have observed to date (CP-AMPAR accumulation, switch in group I mGluR function, altered CB1R tone; [23], [27], [35]) appear to occur in most MSN. This lack of heterogeneity may be because these adaptations appear only after a month or more of withdrawal [28], and therefore are quite temporally removed from the initial effects of cocaine on D1 and D2 receptor expressing subpopulations. Based on these findings, it seems unlikely that the NMDAR plasticity described here is specific to a particular MSN subpopulation, although this should be tested in the future. Related to this issue, it is important to note that segregation of D1 and D2 receptors in MSN of the NAc is incomplete, that there are other anatomical distinctions that contribute importantly to MSN diversity, and that DA receptor subtypes other than D1 and D2 contribute to dopamine transmission [60].

In conclusion, our study is the first to explore functional aspects of cocaine-induced plasticity at the level of individual dendritic spines in the NAc. Our results demonstrate a dramatic restructuring of NMDAR-mediated Ca^2+^ signaling in some NAc spines after prolonged withdrawal from extended access cocaine self-administration. While several possible interpretations of our data exist, we suggest that withdrawal from this cocaine regimen is accompanied by the emergence of a population of NMDAR-lacking spines. It will be important for future studies to examine the connectivity and AMPAR complement of these spines, particularly in light of withdrawal dependent increases in synaptic CP-AMPAR levels.
